# Report on the Use of Sulphate of Quinine, in Miasmatic Diseases of the South

**Published:** 1846-02

**Authors:** W. H. Van Buren

**Affiliations:** Late of the Medical Staff, U. S. Army, Prosector to the Professor of Surgery, University of the City of New York


*Report on the use of Sulphate of Quinine, in Miasmatic Diseases of the South.* By W. H. Van Buren, M. D., late of the Medical Staff, U. S. Army, Prosector to the Professor of Surgery, University of the City of New York.—The following "Report," now published by permission of the Surgeon General of the Army, to whom it was originally made, was furnished in compliance with instructions issued to the Officers of the Medical Staff, in the month of August, 1843.

To those of the profession who are not familiar with the diseases of our southern country the opinions thus imperfectly set forth may appear extravagant and ill-judged. If success, however, is the test of practice, the Archives of the Surgeon General's Bureau will demonstrate their propriety, endorsed as they are by the approval of a majority of the most experienced army surgeons who served in Florida. As I am no longer one of their number, I may assert without impropriety that to the Medical Corps of the Army belongs the credit of fitst testing this practice extensively on our continent, and establishing its superiority.

Whether large doses of quinine are as well suited to Miasmatic diseases occurring in the North and West, as at the South, remains to be proved. Of this fact, however, I am convinced, that long exposure to miasmatic poison begets a tolerance of the remedy beyond that possessed by the resident of a healthy district. Since the "Report" was written I have tested the quinine in large doses in numerous instances in the city of Washington, D. C., and its immediate vicinity, and I have found no reason to alter the opinions already expressed.

*New York*, December, 1845. W. H. V. B.

In answer to your '*Circular*' of the 14th ult., I have the honor to submit the following reply.

My experience in the use of large doses of sulphate of quinine, has been acquired since the commencement of my service in Florida, now more than two years ago. During the whole of this period I have been on duty, and, for the greater part of it, in charge of a command exceeding four companies of U. S. troops. On a rough estimate, I may safely say that I have treated more than 1000 cases of disease of the country up to the present time.

During the first four months of my service in Florida, I was in the habit of using quinine in doses varying from two to twenty grains, according to my judgment at that time. My authority, then, for the use of the medicine in large doses, was the experience of the French Army Surgeons in Africa, and the experiments of M. Piorry and others in Paris in 1838-39. Afterwards, by the experience of Surgeon B. F. Harvey, U. S. Army, I was induced to employ large doses much more generally, and since May, 1841, I hade rarely used less than from ten to twenty grains at a dose, when I wished to secure the full and specific effects of the remedy.

I have invariably employed the sulphate of quinine (super-sulphate when in solution,) procured from the Medical Purveyors of the Army, and, to the best of my knowledge, it has always been of a good quality.

My present experience in the use of the medicine suggests the following mode of practice, which, since its adoption, I have had no occasion to change; on the contrary, its superiority has been uniformly more strongly confirmed.

In *Simple Intermittents* of every variety I am in the habit of administering 15 to 20 grains of quinine, most generally in solution, from six to twelve hours before the chill is expected; always making allowance, when necessary, for its *anticipation*. In *pure ague and fever*, when the derangement of the nervous system has as yet produced no *visceral* or *glandular* disease, I use no '*preparatory medicines*,' believing that thus far the disease lies wholly in the nervous system,—that other symptoms are but consequences of its irregular action,—and, that 'preparatory medicines,' when not positively indicated, are not only unnecessary, but decidedly injurious.

In about two-thirds of the cases thus treated, I have found it unnecessary to *repeat* the dose in order to effect a cure, and I am unable at present to remember an instance in which a *third* dose has been required. I believe that when the proper moment can be exactly ascertained in which to administer 15 or 20 grains of quinine, one dose will prevent the recurrence of a chill in ninety out of a hundred cases, —and that when the disease is thus cut short on its first attack, it is far less liable to recur at a future period.

In *Remittent Fevers*, I am in the habit of combating the paroxysms according to the indications in each case,—rarely by venesection or emetics, most frequently by mercurial cathartics, acidulated drinks and the *free use of cold water to the surface of the body.* The moment that the skin becomes moist, without particular regard to the state of the pulse, I give from 20 to 25 grains of quinine, according to the severity of the case, the constitution of the patient, and the general type of disease at the time,—and repeat it in from four to eight hours. This treatment will in most cases prevent a return of the paroxysm. The most effectual remedies for *gastric irritability* I have found to be a strong infusion of capsicum, and *ice*, held in the mouth, or swallowed in bulk. It has been necessary, however, to administer the quinine whilst ten or twelve cups were drawing on the abdomen, in order to prevent its rejection by the stomach, and, in other cases, to secure its action by the application of 60 or 80 grains to a blistered surface on the epigastrium. If the doses mentioned above can be retained, and absorbed by the stomach, the fever cannot continue.

In *Malignant Intermittents*,—the "*Fièvres Intermittentes Pernicieuses*," of Alibert,—the prompt and decided use of the remedy in the same doses, is successful with equal certainty. In these cases, however, it is necessary to keep up its action for a somewhat longer period than in simple remittents.

In what is called "Congestive Fever," which most frequently occurs as a *Malignant Intermittent of one paroxysm*, I have no precise limit to the use of the remedy. Forty grains, if retained and digested by the stomach, will, I believe, secure its full effect for a longer or shorter period; and generally in ten or fifteen hours reaction comes on—if at all. With nerve and decision in the employment of quinine in full doses, in connexion with stimulants (brandy and capsicum,) and external stimulants (of which the best are aq. ammonia fort. and the decoction of cantharides in sp. terebinth.,) used with the necessary energy and perseverance—I consider this disease—the most severe of an endemic form that occurs in our Southern country—to be perfectly manageable when taken in time by a physician of judgment. Calomel is valuable as a cathartic, and, in certain cases, to allay gastric irritability;—but glisters, especially those of a stimulating character, and if necessary containing quinine, act more rapidly, and in these cases there is rarely time to secure the specific effects of mercury.

In Yellow Fever I regret to say that I have never seen any decided and permanent good effects from the use of quinine—though it was employed in doses of every size in a number of cases at this post, in the autumn of 1841. The trial of the medicine at that time was not decisive—but the results were far from satisfactory. With regard to the pathology of this disease, I have been induced to believe that its *ultimate cause* induces a peculiar action of the liver, which results in a vitiated secretion from this organ, of the character of an animal poison; this, passing into the blood, produces an *alteration of that fluid*, and, as a consequence, of all the secretions, and thus gives rise to the peculiar condition of the nervous system, which characterizes the disease. When the kidneys cease to act, and the *urea* is no longer eliminated from the blood it acts upon the brain as a narcotic poison, as is proved by its effects where the chemical preparation is taken into the stomach. Why may not the elements of the bile, chemically altered, produce an analogous effect? If it were not unconnected with the subject, further evidence might be adduced, which would, I think, give some plausibility to this view of the pathology of Yellow Fever; I have introduced it here to explain, if possible, why quinine does not act as energetically in this as in other forms of fever of a similar character: it is, that the primary seat of the disease in Yellow Fever is the *blood*—and not the nervous system, as in miasmatic diseases—derangement of the nervous system being the *consequence*, and not the *cause* of the disease.

In enlargement of the spleen, as a sequela of Intermittent Fevers, I have never yet failed to effect a cure by large doses of quinine—even in cases where other treatment had failed. The following case will show the mode of its administration.

At Fort King, E. F., in December, 1842, a boy of nine years of age, was brought to me with an enormously enlarged spleen, of eighteen months' duration; he had been under treatment without benefit for some time previously, I presume by the ordinary means. His skin was dry and sallow, appetite depraved, bowels irregular, and spirits entirely gone; no pain on pressure over either liver or spleen. At first sight I thought the boy was dropsical, from the unusual size of his belly, but, on examination, fully one half of the abdominal cavity was found occupied by the spleen, which was hard and well defined in its outlines. I prescribed gr. ss. hydrarg. chlor. mitis, three times a day, which was continued for ten days; at the same time a blister was applied over the spleen, and dressed with sixty grains quin. sulph. pulv., and every other morning twenty-five grains of the same were given internally (in pills of five grs. each) at once, for four successive doses; the fifth dose of twenty-five grains was given after an interval of three days, and the sixth dose four days later. This was all the medicine used, and at the end of the month the spleen was reduced to its natural size, and the child was in perfect health—and so continues at the present time. The most striking circumstance in this case was, that at no time during the treatment could I detect any *cerebral* symptoms from the quinine; in fact its only evident effect was the diminution of the spleen. Of its mode of action in cases of this kind I can afford no explanation. I have invariably observed that children bear large doses of quinine even better than adults. About this time I gave an infant six weeks old *five grains*, stopping its ague at once, and without a bad symptom.

I have seen M. Piorry, in the Hospital de la Pitie, at Paris, after marking out the outlines of an enlarged spleen on the skin of the abdomen with lunar caustic, give twenty-five grains of quinine to the patient, and on examination, *two hours afterwards*, assert that he detected a decided diminution in its size. I did not satisfy myself, however, that this was actually the case—perhaps from want of skill in *palpation*.

In *Masked Intermittents*, I am also convinced of the superior utility of large doses of the medicine. Intermittent hemicrania is the most obstinate form of miasmatic disease that I have encountered.

I have used the sulphate of quinine, experimentally, to a limited extent, in other forms of disease besides those enumerated : in chronic hepatitis, epilepsy, amaurosis, and mania—but without any results of interest. I am prepared to believe, however, that all functional diseases of the nervous system are to some extent under the influence of this medicine, and that it will *cure* any disease of an intermittent character.

I have given twenty grains of quinine on two occasions during *frank open fever*, with hot skin and excited pulse—when alarmed at its long continuance, and fearful that it would result in congestion ; in both cases it acted apparently as a *nervous and arterial sedative*, being followed by relaxation of the skin and falling of the pulse to the natural standard.

I have never seen bad effects from the use of large doses of the medicine, in any stage of fever, though I am not prepared to use it indiscriminately where there is cerebral excitement.

In smaller doses, under the same circumstances, I have been repeatedly impressed with the idea that it increased the febrile symptoms, and failed in producing a sedative, or equalizing effect.

I believe that a full dose of quinine is from fifteen to twenty-five grains, according to the impressibility of the patient ; and that, as is the case with many other medicines, the effect of a smaller, or fractional dose, is very different from that of a full dose ; and that, where the full impression of the remedy is required, it is more economical, as well as more philosophical, and far more likely to be immediately successful, to administer ℈j at once, at the proper time, than gr. ij. every two hours, for three or four days, or longer.

In two instances, after administering grs. xx. of quinine, too late, apparently, to prevent the access of a paroxysm of fever, I have witnessed a singular condition of the system, which seemed like a struggle between the remedy and the disease for mastery over the nervous system ; there was great oppression of breathing, restlessness, and nervous depression ; trembling and chattering of the teeth, unaccompanied by any sensation of coldness, and suppressed and struggling pulse. In both of these cases—the one a healthy lady with one previous chill, the other a soldier after one paroxysm of simple remittent —the remedy apparently gained the victory after the lapse of half an hour, and no fever followed ; from which circumstance I infer that the symptoms enumerated did not constitute a chill. A repetition of the dose was not required in either case, and they both convalesced without a bad symptom.

I have seen one case of *partial loss of hearing* which followed the use of *two grain* doses of quinine for a period of two weeks ; one case of tinnitus aurium, of eleven months' standing, now under treatment, and apparently incurable, attributed to the same cause ; and I have also under treatment a case of amblyopia, of three weeks duration, and quite intractable, which followed the use of *four grain doses* every four hours for a week, for malignant intermittent fever, before my arrival at this post.

I have never yet witnessed any of these results from the use of large doses, and, in fact, with the exception of the temporary aggravation of a chronic dysentery, I have never seen any bad effects from them. In dysenteric cases, I attribute the aggravation of the symptoms more to the sulphuric acid of the solution than to the sulphate of quinine; and accordingly use the medicine, when required in such cases, in the form of pills, combined with sulphate of morphine.

With regard to the *modus operandi* of quinine, I believe that in fractional doses (from gr. ss. to gr. xv.) it acts upon the nervous system as a *direct and peculiar stimulant*, with an ultimate paroxysmal effect. In full doses (gr. xv. and upwards,) its action varies with the condition of the system; in the dynamic stage of misasmatic fevers my impression is that its immediate action is that of a *sedative;* in the adynamic stage of fevers—or in other words, after the resistance of vitality has been overcome by the cause of the disease, either entirely and permanently, as in congestive fever, or temporarily, as in remittents or intermittents—the action of the remedy in large doses, when it produces any effect, is, uniformly and certainly, to restore a healthy tone to the nervous system, and to *stimulate specifically the powers of life* in the most decided manner. Its effects upon the circulation I believe to be altogether *indirect*—one of the consequences of its *equalizing* influence on the human system,—and this rationale explains in some degree its varying action in different states of the system.

It is possible that the sulphate of quinine may exercise an influence over the circulation, directly, by some peculiar action an the *spleen*—admitting that this organ acts the part of a "governor" to the circulation, or a *diverticulum* by which the distribution of the blood is regulated. This would afford some idea of its mode of action in reducing an enlarged spleen to its natural size.

The action of the sulphate of quinine, and still more of the supersulphate, seems to be uniformly slightly irritating to the mucous lining of the intestines—more particularly of the colon and rectum. Beyond this I have no evidence of its unfavorable influence, as has been asserted, upon diseases of the bowels. On the contrary it has so happened that since the frequent use of large doses of the medicine, I have encountered fewer and less severe cases of dysentery than formerly.

With regard to the *climate of Florida*, a subject upon which there has been recently much debate, it is my opinion that, by comparison with other sections of our country, it may be considered as ordinarily healthful. Its acute diseases are remarkably uniform in their character, and, as for a large proportion of them, there is fortunately a remedy—one of the most certain in its action that the materia medica can afford. Its long continued heat, the greater comparative coolness of the nights, and the constant humidity of the atmosphere, have a tendency to produce miasmatic fever, diseases of the liver and digestive organs, and to dispose the skin to ulcerations. I have observed also among the older residents an unusual tendency to diseases depending upon the deposit of accidental tissues, especially of a malignant character, such as carcinomatous ulcers and tumors, and fungus hæmatodes,—always, however, excepting scrofula. In this city hydrocele is of remarkably common occurrence—a large proportion of the natives, and old inhabitants, to my knowledge being affected with it. Whether this is the result of local causes, or produced solely by the climate, I am not prepared to say.

The relation between local causes of disease, and epidemic and endemic influences, is as mysterious here as elsewhere.

I have never seen a case of disease of the liver or spleen, which could—on the most remote evidence—be attributed to the use of quinine, either in large or small doses.

Of the qualities of the sulphate of quinine as a *pure tonic in small doses* I have received an unfavorable impression, in the debility following diseases of this climate. Used in this manner I believe that it acts only as a direct and peculiar permanent stimulant, and that it has a greater tendency to induce general irritability of the system than other and more simple vegetable tonics, with a decidedly unpleasant effect in many instances upon the mucous membrane of the intestines and the brain.

I have never recognized its action upon the liver.

After stopping an intermittent fever by a full dose of quinine, unless the system requires decided support, I am not in the habit generally of administering tonics; and I have never witnessed more satisfactory cases of prompt and uninterrupted convalescence than while pursuing this plan of practice. In cases which required a supporting treatment, the indigenous tonics of the country—the prunus Virginiana and cornus Florida—have proved to be valuable medicines.

According to my observations the unpleasant effects of quinine, in producing ringing in the ears, &c., &c., are more constant and severe in proportion to the smallness of the dose employed. I have seen many patients excessively annoyed by doses of two grains, frequently repeated, who have afterwards, under similar circumstances, taken twenty grains with comparative impunity. The medicine, in solution, has also a greater tendency to produce these symptoms than in pilular form ; still the solution is more immediate and efficient in its action.

I cannot leave this subject without remarking the striking rarity of organic disease as the result of fevers, &c., in this section of the country, in comparison with others, where the same class of diseases prevails.

Dropsies and chronic affections of the liver and spleen are exceedingly rare, taking into consideration the very general prevalence of miasmatic fevers, and acute diseases of the climate,

I have the honor to remain, &c.,

Wm. H. Van Buren, M. D.,
Asst. Surgeon, U. S. A.

*St. Francis Barracks, St. Augustine, E. F., September 10th, 1842.*

*N. Y. Journ. of Med.*

